# Covert macrovascular disease and early outcome after ischemic cerebrovascular events

**DOI:** 10.1007/s00392-026-02947-x

**Published:** 2026-06-08

**Authors:** Priyanka Boettger, Linda Vollmann, Jamschid Sedighi, Hassan Hassan, Heinz Frederik Noll, Pascal Bauer, Tobias Braun, Michael Buerke, Heidrun Kraemer-Best, Martin Juenemann, Omar Alhaj Omar, Samuel Sossalla

**Affiliations:** 1 Department of Cardiology, Angiology and Critical Care Medicine, Justus Liebig University, Klinikstrasse, 33 35392 Giessen, Germany; 2 Department of Neurology and Neurological Intensive Care, Justus Liebig University, Giessen, Germany; 3 Department of Cardiology, Angiology and Critical Care Medicine, St. Marien Hospital, Siegen, Germany; 4 Department of Cardiology, Campus Kerckhoff of the Justus-Liebig-University, Bad Nauheim, Hessen Germany; 5 Cardio-Pulmonary Institute (CPI), Giessen, Hessen Germany

**Keywords:** Ischemic stroke, Transient ischemic attack, Covert macrovascular disease, Non-stenotic carotid plaque, Embolic stroke of undetermined source, Vascular vulnerability, Functional outcome

## Abstract

**Background:**

Covert cerebrovascular disease is traditionally defined as microvascular brain injury. Whether non-culprit macrovascular abnormalities below conventional stenosis thresholds represent a clinically relevant form of vascular vulnerability remains unclear. We investigated the prevalence and prognostic significance of covert macrovascular disease (CMVD) in acute ischemic stroke and transient ischemic attack (TIA).

**Methods:**

In this prospective observational cohort, consecutive patients admitted with ischemic stroke or TIA underwent standardized vascular imaging. CMVD was defined as non-culprit macrovascular pathology not meeting TOAST criteria for large-artery atherosclerosis, including non-stenotic carotid or vertebral plaques (< 50%), plaques outside the infarct-supplying territory, aortic arch atheroma (≥ 2 mm), or non-hemodynamically relevant vascular elongation or kinking. Lesions were adjudicated by blinded experts. The primary outcome was favorable functional status at discharge (modified Rankin Scale [mRS] 0–2). Multivariable logistic regression adjusted for demographic, clinical, and vascular risk factors.

**Results:**

Among 714 patients (mean age 72 ± 9 years; 43% women), CMVD was present in 271 (37.9%; 95% CI, 34.4–41.6). CMVD prevalence varied by stroke etiology, highest in large-artery atherosclerosis (56%) and cardioembolic stroke (41%), intermediate in ESUS (35%), and lowest in small-vessel occlusion (21%). Patients with CMVD had higher admission stroke severity and lower rates of favorable functional outcome at discharge (47.1% vs. 63.2%; p = 0.001), as well as higher in-hospital mortality (6.3% vs. 2.7%; p = 0.049). CMVD remained independently associated with unfavorable outcome (adjusted OR 1.82; 95% CI, 1.18–2.81; p = 0.006) and an increased risk of recurrent ischemic stroke during follow-up (HR, 1.84; 95% CI, 1.05–3.20).

**Conclusions:**

Covert macrovascular disease is common in acute ischemic cerebrovascular events and independently predicts worse early functional outcome. These findings support CMVD as a clinically meaningful marker of systemic vascular vulnerability beyond culprit stenosis.

**Graphical Abstract:**

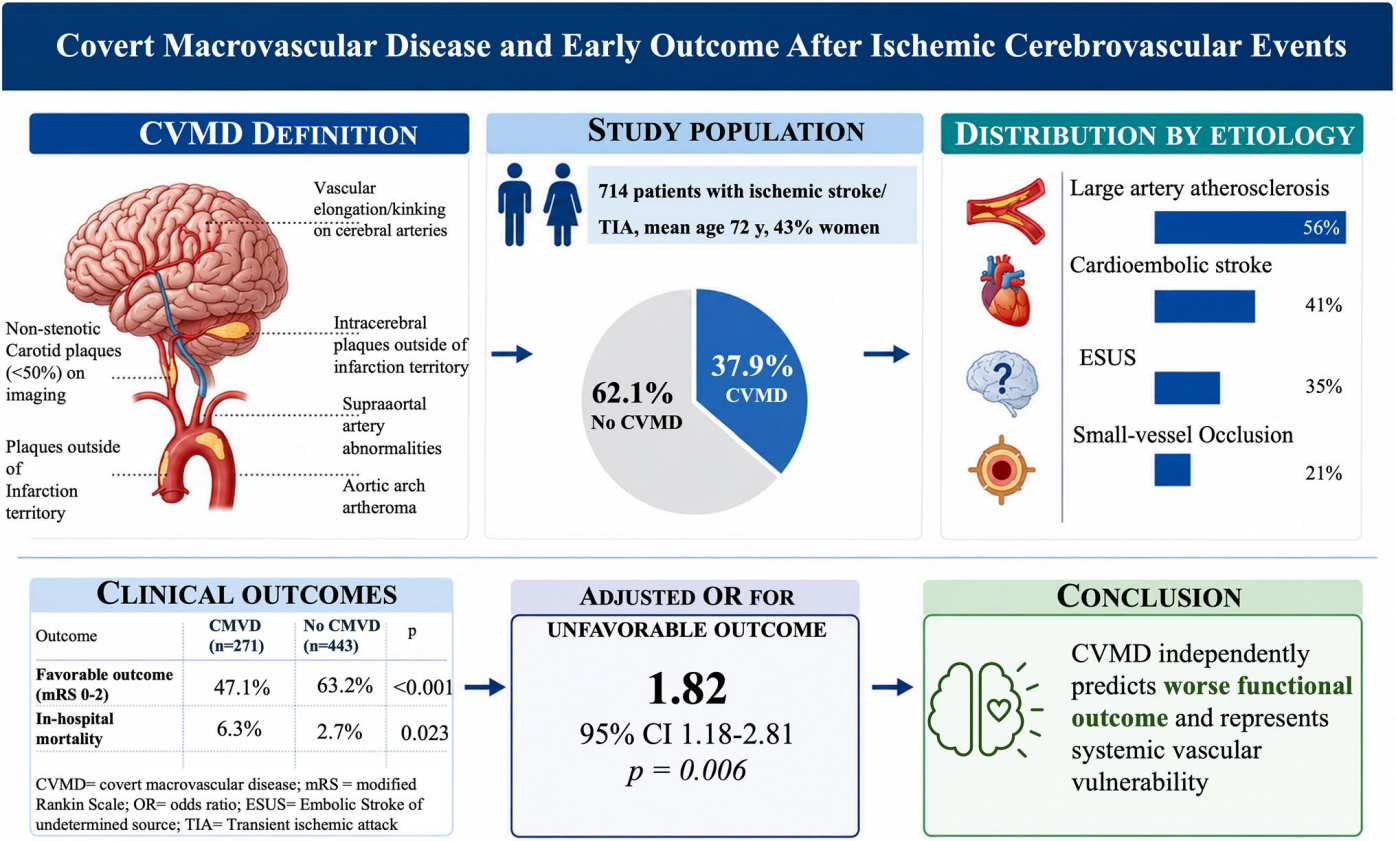

## Introduction

Covert cerebrovascular disease traditionally refers to subclinical microvascular brain injury, including white-matter hyperintensities and cerebral microbleeds, which are associated with increased stroke risk, cognitive impairment, and reduced neurological resilience, but do not capture potential contributions from subclinical macrovascular pathology [1, 2]. Yet cerebrovascular vulnerability extends beyond the microvascular domain. Increasing evidence suggests that macrovascular abnormalities which do not meet conventional criteria for culprit lesions, such as non-stenotic carotid plaques, contralateral or non-territorial atherosclerosis, and aortic arch atheroma, may influence cerebral perfusion, embolic propensity, and functional recovery after ischemic stroke [1, 3–6]. Classical stroke taxonomy has long assumed that large-artery atherosclerosis becomes clinically relevant only when stenosis ≥ 50% affects the artery supplying the infarct, thereby fulfilling criteria for a “culprit lesion” [7, 8].

However, modern imaging techniques reveal that even non-culprit or non-stenotic plaques can exhibit high-risk features including intraplaque hemorrhage, ulceration, and lipid-rich necrotic cores [9–12].

These findings challenge the traditional stenosis-based paradigm and suggest that atherosclerosis acts as a systemic process in which sub-threshold macrovascular abnormalities may reflect an underlying state of vascular vulnerability [13–15]. Whether covert macrovascular disease represents a distinct clinical entity or rather a composite phenotype of diffuse vascular injury remains incompletely understood.

Such lesions are frequently observed in embolic stroke of undetermined source (ESUS), where no high-grade stenosis or major cardioembolic source is identified [7, 16–18]. Yet their prognostic implications remain insufficiently characterized [11]. Prior research has focused on etiological attribution or plaque morphology, while little is known about how covert macrovascular disease interacts with systemic metabolic factors, such as prediabetes, obesity, and central adiposity to influence stroke severity or functional outcome [19, 20]. Metabolic dysregulation may amplify endothelial dysfunction and vascular inflammation, thereby accelerating subclinical arterial injury [21–24]. Refining our understanding of how sub-threshold macrovascular abnormalities that do not fulfill conventional culprit-lesion criteria relate to systemic vascular comorbidity may improve prognostication and inform secondary prevention beyond traditional dichotomies of stenotic versus non-stenotic disease [25–27]. In this prospective study, we therefore investigated the prevalence and prognostic relevance of covert macrovascular disease in patients with acute ischemic stroke. We hypothesized that covert macrovascular disease represents a marker of systemic vascular vulnerability and is associated with increased stroke severity and poorer early functional outcomes.

## Methods

### Study design and population

This prospective observational cohort study included consecutive patients admitted to the Stroke Unit of the academic hospital within one year with suspected diagnosis of acute stroke or transient ischemic attack (TIA). Patients with hemorrhagic stroke or hospitalization shorter than 24 h were excluded. The study adhered to the Declaration of Helsinki and was approved by the Ethics Committee of the Medical Association of Westphalia-Lippe (file number: 2015–091-fS). Written informed consent was obtained from all participants or their legal representatives.

Demographic data, vascular risk factors, comorbidities, and laboratory parameters were collected systematically at admission. Stroke severity was assessed using the National Institutes of Health Stroke Scale (NIHSS) at presentation and discharge [28, 29], and functional outcome was evaluated with the modified Rankin Scale (mRS) at discharge [30, 31]. Both instruments were applied according to current AHA/ASA standards [32].

### Neuroimaging and stroke classification

All patients underwent brain imaging (MRI or CT) to assess for acute ischemia and to classify cerebrovascular events. Ischemic stroke was defined by the presence of imaging-confirmed infarction or a corresponding clinical syndrome, whereas transient ischemic attack (TIA) was defined by transient neurological symptoms without evidence of acute infarction on imaging.

Stroke subtype was classified according to the Trial of Org 10172 in Acute Stroke Treatment (TOAST) criteria [8] and, when appropriate, the Embolic Stroke of Undetermined Source (ESUS) definition [7], based on a standardized diagnostic work-up.

Extracranial and intracranial vascular imaging was performed using carotid duplex ultrasonography and/or computed tomography angiography (CTA) or magnetic resonance angiography (MRA), according to clinical indication. Cardiac and aortic embolic sources were evaluated using transthoracic echocardiography (TTE) and, when clinically indicated, transesophageal echocardiography (TEE). Imaging findings were interpreted by experienced investigators, with interdisciplinary adjudication by cardiology, neurology, and neuroradiology specialists using standardized criteria. A stenosis of ≥ 50% within the vascular territory supplying the infarct was defined as a culprit lesion consistent with large-artery atherosclerosis [7, 8, 33]. Pre-specified subgroup analyses were performed according to stroke subtype and metabolic phenotype.

### Definition of covert macrovascular disease

Covert macrovascular disease (CMVD) was defined as the presence of atherosclerotic or structural vascular abnormalities not meeting conventional criteria for culprit large-artery atherosclerosis, in accordance with prior studies on non-culprit and systemic atherosclerosis [6, 34, 35]. CMVD included the following pre-specified lesion categories:

- Non-stenotic atherosclerotic plaques (< 50% luminal narrowing) in the carotid or vertebral arteries, irrespective of laterality.
- Plaques located outside the infarct-supplying vascular territory, including contralateral carotid or aortic segments without direct embolic continuity.
- Aortic arch atheroma ≥ 2 mm or complex plaques ≥ 4 mm (ulcerated or mobile) identified by TEE or CTA.
- Elongation, kinking, or aneurysmal dilation of major extracranial vessels without hemodynamic significance.

Aortic arch plaque thickness was assessed on TEE or CTA using the maximum plaque thickness perpendicular to the vessel wall. To improve reproducibility, aortic lesions, particularly plaques close to the 2-mm threshold, were reviewed by experienced investigators blinded to clinical outcome, and discrepant or borderline findings were resolved by consensus.

Lesions were adjudicated using standardized criteria by consensus between a vascular neurologist and a cardiologist, both blinded to clinical outcomes. Findings were classified as covert if they did not fulfill TOAST criteria for culprit large-artery atherosclerosis, irrespective of laterality, provided that luminal narrowing remained below the conventional ≥ 50% stenosis threshold and no definite causal relationship to the index infarct could be established. Given the heterogeneous nature of CMVD, lesion-specific analyses were additionally performed for major CMVD components, including non-stenotic carotid plaque, aortic arch atheroma, vertebrobasilar plaque, and non-hemodynamically relevant elongation or kinking, to explore their individual associations with early functional outcome.

### Outcome measures

The primary outcome was early functional status at discharge, defined as a modified Rankin Scale (mRS) score of 0 to 2 [36, 37]. Secondary outcomes included stroke severity at admission, assessed by the National Institutes of Health Stroke Scale (NIHSS), and in-hospital mortality. Associations between CMVD and systemic vascular risk factors, including prediabetes, obesity, and atrial arrhythmia, were analyzed separately, including their role as predictors of CMVD and modifiers of clinical outcome [16, 19, 38]. Pre-specified subgroup analyses were performed according to stroke subtype (cardioembolic, ESUS, large-artery atherosclerosis, and small-vessel occlusion) and metabolic phenotype. In addition, vessel-level analyses were conducted to assess the association between non-stenotic carotid disease and ipsilateral stroke.

### Long-term follow-up and survival analysis

Long-term follow-up for recurrent ischemic stroke was obtained from clinical records and registry-based outcome assessment. Time-to-event analyses were performed using Kaplan–Meier estimates and compared with the log-rank test. Cox regression was used to estimate univariable hazard ratios for recurrent stroke.

### Statistical analysis

Continuous variables are presented as mean ± standard deviation (SD) or median with interquartile range (IQR) (Shapiro–Wilk test). Categorical variables are expressed as counts and percentages. Comparisons were made using the chi-square or Fisher’s exact test for categorical data and t-test or Mann–Whitney U test for continuous data. Multivariable logistic regression identified independent predictors of unfavorable outcome (mRS > 2) after adjustment for age, sex, stroke subtype, NIHSS at admission, and major vascular risk factors. Associations between CMVD and metabolic markers (e.g. HbA₁c, waist-to-hip ratio) were evaluated by linear and logistic regression. All analyses were performed with SPSS Statistics 28.0 (IBM Corp., Armonk, NY). Two-sided p values < 0.05 were considered statistically significant.

## Results

### Study population

**Fig. 1:**
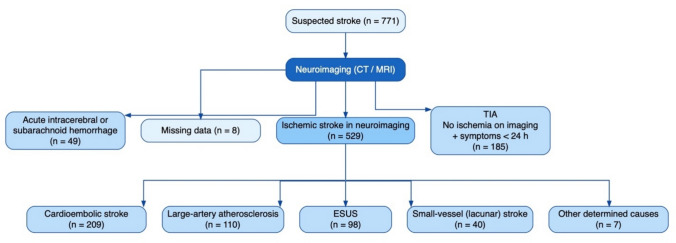
Patient flow and classification of cerebrovascular events. Among 771 individuals presenting with suspected stroke, all underwent neuroimaging with CT or MRI. Imaging results identified transient ischemic attack (TIA; no ischemia on imaging and symptom resolution < 24 h), intracerebral or subarachnoid hemorrhage (ICH), cases with missing diagnostic data, and ischemic stroke. Patients with imaging-confirmed ischemic stroke (*n* = 529) were subsequently classified into etiologic subtypes: cardioembolic stroke (CE), large-artery atherosclerosis (LAA), embolic stroke of undetermined source (ESUS), small-vessel occlusion (SVO), and other determined causes

A total of 714 patients with acute ischemic stroke or TIA were included. The mean age was 72 ± 9 years, and 43.3% were women. According to the TOAST classification, 209 patients (29.3%) had cardioembolic stroke, 110 (15.4%) large-artery atherosclerosis, 40 (5.6%) small-vessel occlusion, 163 (22.8%) cryptogenic stroke, and 7 (1.0%) other determined etiologies. Among cryptogenic strokes, 98 patients fulfilled ESUS criteria. TIA accounted for 185 cases (25.9%) (Fig. 1). The median NIHSS score at admission was 7 (IQR, 3–11), and the median mRS at discharge was 2 (IQR, 1–4). Baseline characteristics are summarized in Table 1.

**Table 1:** Baseline characteristics and clinical outcomes in patients with and without CMVD

Characteristic	Total (*n* = 714)	No CMVD (*n* = 443)	CMVD (*n* = 271)	*p*-value
Age	72 ± 9	69 ± 10	74 ± 8	< 0.001
Female sex	309 (43.3)	213 (48.1)	96 (35.4)	0.002
Hypertension	537 (75.2)	311 (70.2)	226 (83.4)	0.001
Diabetes mellitus	213 (29.8)	119 (26.9)	94 (34.7)	0.041
Prediabetes	236 (33.0)	128 (28.9)	108 (39.9)	0.046
Obesity	334 (46.8)	189 (42.6)	145 (53.5)	0.008
Waist-to-hip ratio	0.95 ± 0.07	0.94 ± 0.07	0.97 ± 0.08	0.011
Atrial fibrillation	163 (22.8)	95 (21.4)	68 (25.1)	0.29
Coronary artery disease	191 (26.8)	99 (22.3)	92 (33.9)	0.002
NIHSS on admission	7 (3–11)	6 (3–9)	8 (4–12)	< 0.001
mRS at discharge	2 (1–4)	2 (1–3)	3 (2–4)	< 0.001
Favorable outcome	408 (57.1)	280 (63.2)	128 (47.1)	0.001
In-hospital mortality	29 (4.1)	12 (2.7)	17 (6.3)	0.049

Continuous variables are presented as mean ± standard deviation (SD) or median (interquartile range, IQR) as appropriate. Categorical variables are expressed as number (percentage)

### Prevalence of covert macrovascular disease

CMVD was present in 271 of 714 patients (37.9%). The most frequent manifestation was non-stenotic carotid disease (NSCD), including 195 patients with non-stenotic carotid plaque (*n* = 195; 27.3% of the overall cohort), followed by aortic arch atheroma ≥ 2 mm (*n* = 68; 9.5%) and elongation or kinking of major extracranial arteries (n = 48; 6.7%). Complex aortic plaques ≥ 4 mm or ulcerated were uncommon (*n* = 21; 3.0%). More than half of CMVD lesions (52%) were located outside the infarct-supplying vascular territory. The prevalence of CMVD differed significantly across etiologic stroke subtypes (*p* < 0.001) (Table 2). CMVD was most frequent in large-artery atherosclerosis (56%) and cardioembolic stroke (41%), followed by ESUS (35%) and cryptogenic non-ESUS stroke (28%).

**Table 2:** Baseline characteristics by stroke subtype

Characteristic	LAA (*n* = 110)	CE (*n* = 209)	ESUS (*n* = 98)	SVO (*n* = 40)	TIA (*n* = 185)	*p*-value
Age, y	74 ± 8	76 ± 9	70 ± 9	68 ± 8	69 ± 9	< 0.001
Female sex	44 (40.0)	97 (46.4)	38 (38.8)	18 (45.0)	82 (44.3)	0.52
Hypertension	94 (85.5)	163 (78.0)	71 (72.4)	31 (77.5)	138 (74.6)	0.018
Diabetes	39 (35.5)	62 (29.7)	31 (31.6)	11 (27.5)	54 (29.2)	0.56
Dyslipidemia	69 (62.7)	101 (48.3)	45 (45.9)	20 (50.0)	81 (43.8)	0.041
Current smoking	27 (24.5)	36 (17.2)	22 (22.4)	6 (15.0)	34 (18.4)	0.27
BMI ≥ 30 kg/m^2^	38 (34.5)	56 (26.8)	28 (28.6)	11 (27.5)	55 (29.7)	0.49
Prior stroke/TIA	31 (28.2)	46 (22.0)	19 (19.4)	5 (12.5)	37 (20.0)	0.11
Baseline NIHSS	8 (5–12)	12 (8–17)	9 (5–14)	4 (2–7)	2 (1–4)	< 0.001
mRS at discharge	3 (2–4)	4 (3–5)	3 (2–4)	2 (1–3)	1 (0–2)	< 0.001
IV thrombolysis	61 (55.5)	126 (60.3)	62 (63.3)	12 (30.0)	78 (42.2)	0.002
Endovascular therapy	37 (33.6)	92 (44.0)	48 (49.0)	4 (10.0)	0 (0.0)	< 0.001
Any covert macrovascular disease	**62 (56.4)**	**86 (41.1)**	**34 (34.7)**	**8 (20.0)**	**52 (28.1)**	** < 0.001**

Continuous variables are presented as mean ± standard deviation (SD) or median (interquartile range [IQR]), as appropriate. Categorical variables are expressed as number (percentage). P values were calculated using one-way ANOVA or the Kruskal–Wallis test for continuous variables and the χ^2^ test or Fisher’s exact test for categorical variables

For clarity and comparability, the table displays the major predefined cerebrovascular event subtypes. Patients with cryptogenic non-ESUS stroke (*n* = 65) and other determined etiologies (*n* = 7) were included in the overall cohort but are not shown separately owing to small subgroup sizes

*LAA* large-artery atherosclerosis, *CE* cardioembolic stroke, *ESUS* embolic stroke of undetermined source, *SVO* small-vessel occlusion, *TIA* transient ischemic attack, *NIHSS* National Institutes of Health Stroke Scale, *mRS* modified Rankin Scale, *BMI* body mass index

**Fig. 2:**
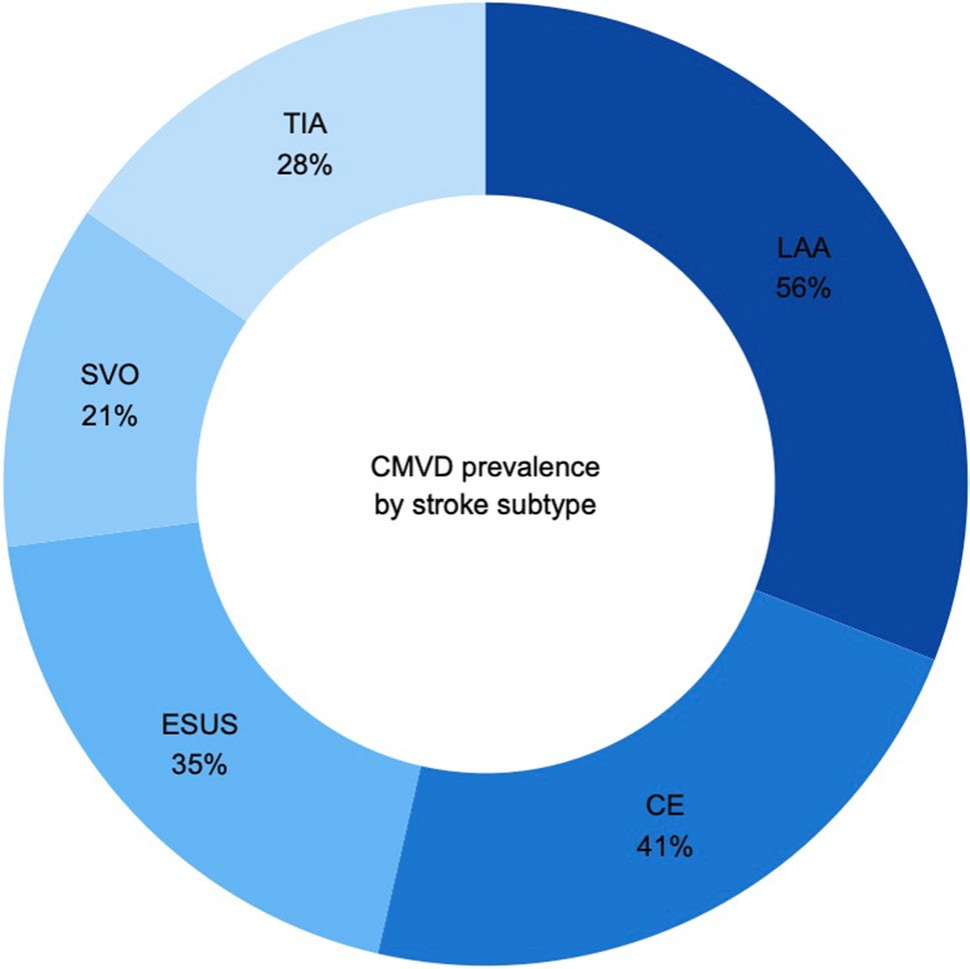
Prevalence of covert macrovascular disease (CMVD) across ischemic stroke subtypes. The prevalence of CMVD was highest in large-artery atherosclerosis (LAA) and cardioembolic stroke (CE), intermediate in ESUS and cryptogenic non-ESUS stroke, and lowest in small-vessel occlusion (SVO) and other determined etiologies. TIA showed a moderate prevalence. Bars indicate the proportion of patients in each etiologic category who met CMVD criteria based on non-stenotic carotid or aortic lesions. Abbreviations: CMVD, covert macrovascular disease; LAA, large-artery atherosclerosis; CE, cardioembolic stroke; ESUS, embolic stroke of undetermined source; SVO, small-vessel occlusion; TIA, transient ischemic attack

**Fig. 3:**
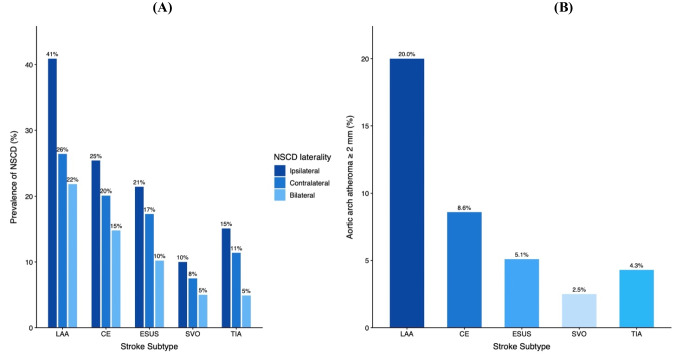
Morphological distribution of covert macrovascular lesions across ischemic stroke subtypes. **(A)** Laterality pattern of non-stenotic carotid disease (NSCD) in patients with NSCD, showing the proportions of ipsilateral, contralateral, and bilateral plaques across stroke subtypes. **(B)** Prevalence of aortic arch atheroma ≥ 2 mm by stroke subtype. Both panels demonstrate that covert macrovascular disease frequently presents as a diffuse, multiterritorial atherosclerotic process rather than a focal abnormality limited to a single vascular territory. Abbreviations: NSCD, non-stenotic carotid disease; LAA, large-artery atherosclerosis; CE, cardioembolic stroke; ESUS, embolic stroke of undetermined source; SVO, small-vessel occlusion; TIA, transient ischemic attack

Lower prevalences were observed in small-vessel occlusion (21%), transient ischemic attack (28%), and other determined etiologies (12%). Compared with all non-LAA subtypes combined, LAA showed a significantly higher CMVD burden (RR 1.95; 95% CI, 1.53–2.49; *p* < 0.001) (Fig. 2). Patients with CMVD were older (74 ± 8 vs. 69 ± 10 years; *p* < 0.001) and had a higher prevalence of hypertension (83% vs. 70%; p = 0.001), diabetes (35% vs. 27%; p = 0.041), and prediabetes (40% vs. 29%; p = 0.046). Waist-to-hip ratio was also higher in the CMVD group (0.97 ± 0.08 vs. 0.94 ± 0.07; p = 0.011) (Table 1 and Fig. 3).

### Morphology of covert macrovascular lesions

Among patients with CMVD, non-stenotic carotid plaques (< 50% stenosis) were the most frequent lesion type (195/271; 72%). Their distribution and morphological characteristics varied across stroke subtypes (Table 3). The prevalence of any NSCD differed significantly across subtypes, ranging from 56% in large-artery atherosclerosis (LAA) and 41% in cardioembolic stroke (CE) to 35% in ESUS, 20% in small-vessel occlusion (SVO), and 28% in transient ischemic attack (TIA) (*p* < 0.001). Ipsilateral NSCD was most common in LAA (41%) and CE (25%), less frequent in ESUS (21%), and rare in SVO (10%) and TIA (15%) (*p* < 0.001). Bilateral plaques were more frequent in LAA (22%) and CE (15%) than in ESUS (10%) and TIA (5%) (p = 0.002). Aortic arch atheroma ≥ 2 mm also varied by stroke subtype, occurring most often in LAA (20%) compared with CE (9%), ESUS (5%), and SVO/TIA (≤ 4%) (*p* < 0.001). Stenosis grade did not differ across subtypes (all p > 0.80). High-risk plaque features, defined as plaque thickness ≥ 3 mm, irregular surface, or ulceration, were observed in 34–44% of lesions but did not differ significantly between subtypes (all p > 0.70).

**Table 3:** Distribution and characteristics of non-stenotic carotid disease (NSCD) by stroke subtype

Descriptor	LAA	CE	ESUS	SVO	TIA	*p*-value
Patients with any NSCD	62 (56.4)	86 (41.1)	34 (34.7)	8 (20.0)	52 (28.1)	< 0.001
Laterality						
Ipsilateral NSCD	45 (40.9)	53 (25.4)	21 (21.4)	4 (10.0)	28 (15.1)	< 0.001
Contralateral NSCD	29 (26.4)	42 (20.1)	17 (17.3)	3 (7.5)	21 (11.4)	0.006
Bilateral plaques	24 (21.8)	31 (14.8)	10 (10.2)	2 (5.0)	9 (4.9)	0.002
Vascular territory						
Aortic arch atheroma ≥ 2 mm	22 (20.0)	18 (8.6)	5 (5.1)	1 (2.5)	8 (4.3)	< 0.001
Vertebrobasilar plaque	8 (7.3)	7 (3.3)	2 (2.0)	1 (2.5)	3 (1.6)	0.14
Stenosis grade of covert lesions						
0–9%	6 (9.7)	9 (10.5)	3 (8.8)	1 (12.5)	4 (7.7)	0.99
10–19%	11 (17.7)	18 (20.9)	7 (20.6)	2 (25.0)	10 (19.2)	0.95
20–39%	33 (53.2)	41 (47.7)	18 (52.9)	4 (50.0)	29 (55.8)	0.83
40–49%	12 (19.4)	18 (20.9)	6 (17.6)	1 (12.5)	9 (17.3)	0.96
Plaque features on CTA						
Plaque thickness ≥ 3 mm	21 (33.9)	25 (29.1)	10 (29.4)	2 (25.0)	13 (25.0)	0.79
Irregular surface	16 (25.8)	22 (25.6)	7 (20.6)	1 (12.5)	9 (17.3)	0.72
Ulceration	7 (11.3)	9 (10.5)	3 (8.8)	0 (0.0)	4 (7.7)	0.87
≥ 1 high-risk feature*	27 (43.5)	35 (40.7)	13 (38.2)	2 (25.0)	18 (34.6)	0.68

Values are presented as number (percentage). Percentages refer to the respective stroke subtype. *P* values were calculated using the χ^2^ test or Fisher’s exact test. *LAA* large-artery atherosclerosis, *CE* cardioembolic stroke, *ESUS* embolic stroke of undetermined source, *SVO* small-vessel occlusion, *TIA* transient ischemic attack, *NSCD* non-stenotic carotid disease. High-risk features include plaque thickness ≥ 3 mm, irregular surface, ulceration, or carotid web

### Clinical and metabolic correlates of covert macrovascular disease

Among the 714 patients included, 271 (37.9%) met criteria for CMVD, whereas 443 (62.1%) had no CMVD (Table 1). Patients with CMVD were older than those without CMVD (74 ± 8 vs. 69 ± 10 years, *p* < 0.001) and less frequently female (35.4% vs. 48.1%, p = 0.002).

Classical vascular risk factors were more prevalent among patients with CMVD. Hypertension (83.4% vs. 70.2%, p = 0.001), diabetes mellitus (34.7% vs. 26.9%, p = 0.041), and prediabetes (39.9% vs. 28.9%, p = 0.046) were more common in the CMVD group. Markers of adiposity were also higher in these patients, including obesity (53.5% vs. 42.6%, p = 0.008) and waist-to-hip ratio (0.97 ± 0.08 vs. 0.94 ± 0.07, p = 0.011). Coronary artery disease was more frequent in patients with CMVD (33.9% vs. 22.3%, p = 0.002), whereas the prevalence of atrial fibrillation did not differ between groups (25.1% vs. 21.4%, p = 0.29). In multivariable logistic regression analysis adjusting for demographic and vascular risk factors, hypertension (adjusted OR 1.74; 95% CI 1.14–2.65) and prediabetes (adjusted OR 1.39; 95% CI 1.02–2.00) emerged as independent predictors of CMVD.

### Stroke severity, early functional outcome, and multivariable analyses

**Fig. 4:**
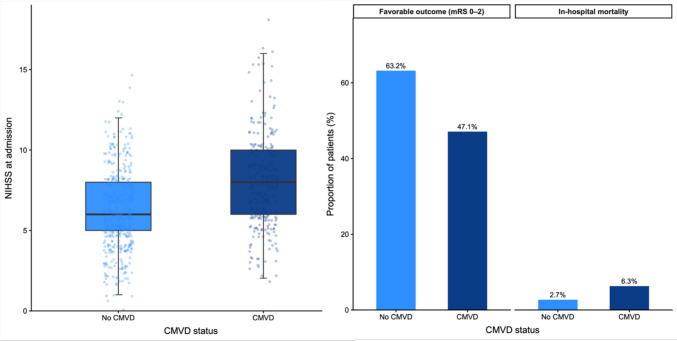
Association of covert macrovascular disease (CMVD) with stroke severity and early outcomes. Panel **A**: Stroke severity at admission, measured by the National Institutes of Health Stroke Scale (NIHSS), was significantly higher among patients with CMVD than those without CMVD. The median NIHSS was 8 (IQR, 4–12) in the CMVD group compared with 6 (IQR, 3–9) in the non-CMVD group (p < 0.001). Boxplots display the distribution, and overlaid diamonds represent the mean ± standard deviation. Panel **B:** Clinical outcomes at discharge differed significantly by CMVD status. Patients with CMVD had lower rates of favorable functional outcome (modified Rankin Scale, mRS 0–2: 47.1% vs. 63.2%) and higher in-hospital mortality (6.3% vs. 2.7%). Data reflect observed proportions from the prospective cohort of 714 patients

Stroke severity at admission differed significantly between groups (Fig. 4A, Table 1). Patients with CMVD presented with greater neurological impairment, with a median NIHSS score of 8 (IQR, 4–12) compared with 6 (IQR, 3–9) in patients without CMVD (*p* < 0.001). Functional outcomes at discharge were also poorer in the CMVD group (Fig. 4B). The median mRS score was 3 (IQR, 2–4) in patients with CMVD versus 2 (IQR, 1–3) in those without CMVD (*p* < 0.001). A favorable outcome (mRS 0–2) was achieved in 47.1% of patients with CMVD compared with 63.2% of those without CMVD (p = 0.001). In-hospital mortality was higher among patients with CMVD (6.3% vs. 2.7%, p = 0.049).

Vessel-level analysis demonstrated that non-stenotic carotid disease (NSCD) was associated with higher odds of ipsilateral stroke in large-artery atherosclerosis (adjusted OR, 2.42; 95% CI, 1.36–4.30; p = 0.003), cardioembolic stroke (adjusted OR, 1.89; 95% CI, 1.07–3.34; p = 0.028), and ESUS (adjusted OR, 1.63; 95% CI, 1.01–2.62; p = 0.046), whereas associations in small-vessel occlusion (adjusted OR, 0.92; 95% CI, 0.38–2.25; p = 0.86) and transient ischemic attack (adjusted OR, 1.28; 95% CI, 0.71–2.33; p = 0.41) did not reach statistical significance (Table 4). The interaction between stroke subtype and NSCD was significant (p = 0.024).

**Table 4:** Association of non-stenotic carotid disease (NSCD) with ipsilateral stroke by subtype

Stroke subtype	Adjusted OR (95% CI) for ipsilateral stroke with NSCD	*p*-value
Large-artery atherosclerosis (LAA)	**2.42 (1.36–4.30)**	**0.003**
Cardioembolic (CE)	**1.89 (1.07–3.34)**	**0.028**
Embolic stroke of undetermined source (ESUS)	**1.63 (1.01–2.62)**	**0.046**
Small-vessel occlusion (SVO)	0.92 (0.38–2.25)	0.86
Transient ischemic attack (TIA)	1.28 (0.71–2.33)	0.41
Global interaction (Subtype × NSCD)	χ^2^ = 11.3, df = 4, p = 0.024	

Model: multivariable logistic regression adjusted for age, sex, NIHSS at admission, hypertension, diabetes, dyslipidemia, and smoking; clustering on patient ID for vessel-level observations

**Fig. 5:**
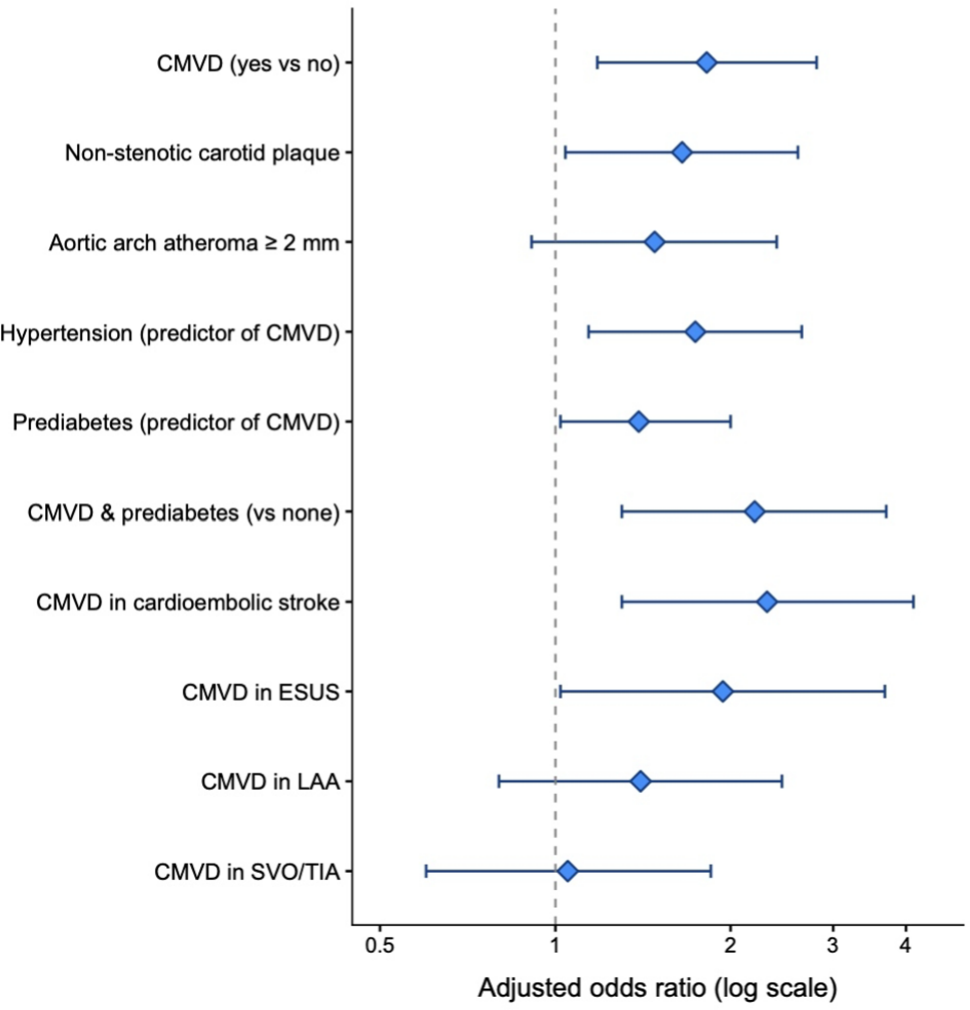
Covert macrovascular disease and early functional outcome after ischemic stroke. Adjusted odds ratios for unfavorable functional outcome associated with covert macrovascular disease (CMVD), lesion characteristics, metabolic factors, and stroke subtypes. Diamonds represent adjusted odds ratios, and horizontal lines indicate 95% confidence intervals. CMVD was independently associated with unfavorable early functional outcome. Non-stenotic carotid plaque showed the strongest lesion-specific association, whereas aortic arch atheroma showed a nonsignificant trend. Associations were strongest in cardioembolic stroke and ESUS, with no significant associations observed in the pooled SVO/TIA subgroup. Abbreviations: CMVD, covert macrovascular disease; ESUS, embolic stroke of undetermined source; TIA, transient ischemic attack

In multivariable logistic regression adjusting for demographic and vascular risk factors, the presence of CMVD remained independently associated with unfavorable outcome (adjusted OR, 1.82; 95% CI, 1.18–2.81; p = 0.006) (Fig. 5). Among individual lesion types, non-stenotic carotid plaques showed the strongest association with poor early outcome (adjusted OR, 1.65; 95% CI, 1.04–2.61; p = 0.034), whereas aortic arch atheroma showed a nonsignificant trend (adjusted OR, 1.48; 95% CI, 0.91–2.40; p = 0.11).

Less frequent or structurally heterogeneous CMVD components, including vertebrobasilar plaque and non-hemodynamically relevant elongation or kinking, showed weaker and less consistent associations with outcome. The association between CMVD and outcome varied across stroke subtypes, with the strongest effects observed in CE (adjusted OR, 2.31; 95% CI, 1.30–4.12; p = 0.004) and ESUS (adjusted OR, 1.94; 95% CI, 1.02–3.68; p = 0.042), whereas no statistically significant associations were observed in the pooled SVO/TIA subgroup, although these subgroup analyses were limited by smaller sample sizes and wider confidence intervals (Fig. 5). Consistent with these findings, among patients with prediabetes, functional independence (mRS 0–2) was achieved in 39% of those with CMVD as compared with 64% of those without CMVD (p = 0.008). Accordingly, the combined presence of CMVD and prediabetes was associated with a numerically stronger adverse effect on early functional outcome.

### Long-term recurrent stroke

**Fig. 6:**
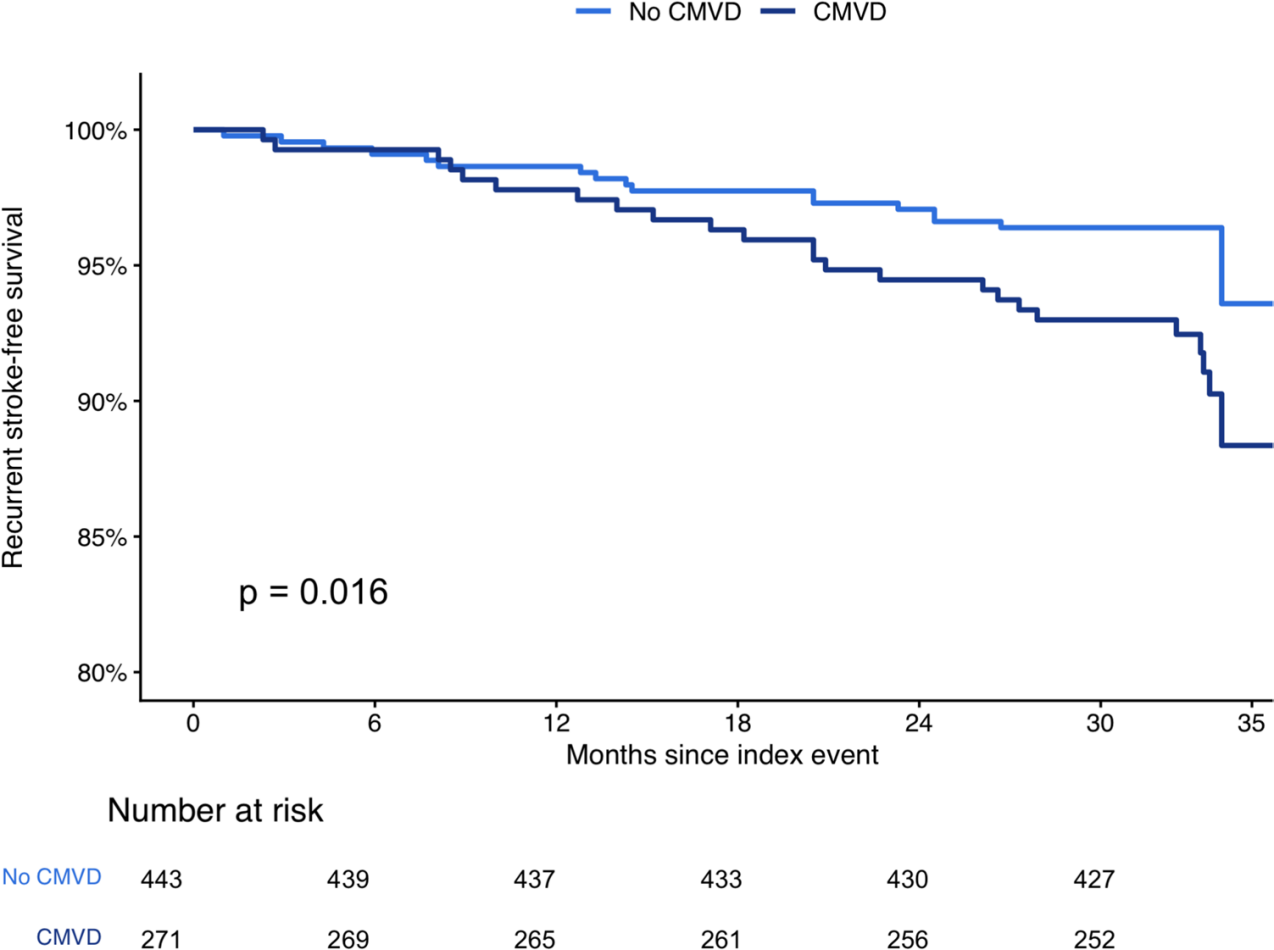
Kaplan–Meier estimates of recurrent stroke–free survival according to covert macrovascular disease (CMVD) status. Patients with CMVD showed a higher cumulative risk of recurrent ischemic stroke during follow-up. Survival curves were compared using the log-rank test. Numbers at risk are shown below the plot

During a median follow-up of 36 months (IQR, 24–36), recurrent ischemic stroke occurred in 46 patients. Kaplan–Meier analysis showed a lower stroke-free survival among patients with CMVD than among those without CMVD (log-rank p = 0.016) (Fig. 6). Stroke-free survival at 36 months was 90.8% in the CMVD group and 95.3% in the non-CMVD group. In univariable Cox regression, CMVD was associated with an increased risk of recurrent ischemic stroke (hazard ratio, 1.84; 95% CI, 1.05–3.20; p = 0.032).

## Discussion

In this prospective cohort of patients with acute ischemic cerebrovascular events, CMVD, defined as macrovascular pathology below conventional culprit-stenosis thresholds, was highly prevalent and independently associated with greater neurologic severity and poorer early functional outcome. These findings suggest that macrovascular pathology beyond conventional stenosis thresholds contributes to cerebrovascular vulnerability and may represent an under-recognized determinant of stroke severity and recovery.

### CMVD as an extension of covert cerebrovascular disease into the macrovascular domain

While covert cerebrovascular disease has classically referred to microvascular injury such as silent infarcts and white-matter hyperintensities, large-scale neuroimaging studies demonstrate that these subclinical lesions are substantially more common than overt stroke and predict increased risk of future stroke, dementia, and mortality [39]. Similar patterns of “silent but significant” pathology are well established in systemic vascular disease: in diabetes and cardiometabolic cohorts, macrovascular complications, including coronary, cerebrovascular, and peripheral artery disease, often develop years before clinical manifestation, with prevalence approaching 40–50% in middle-aged individuals [40–43]. Many of these macrovascular abnormalities remain clinically covert yet substantially increase the risk of myocardial infarction, stroke, and all-cause mortality [43, 44].

Our finding that more than one-third of patients with acute ischemic stroke exhibited CMVD suggests that covert macrovascular pathology is not incidental but reflects a broader systemic vascular milieu. This expands the conceptual framework of covert cerebrovascular disease into the macrovascular domain, indicating that both micro- and macrovascular injury may represent different manifestations of a shared vascular vulnerability state.

### Co-segregation of CMVD with metabolic and cardiovascular risk factors

The strong association between CMVD and hypertension, glycemic dysregulation, obesity, and central adiposity in our cohort aligns with extensive epidemiologic evidence showing that macrovascular and metabolic abnormalities cluster within the same individuals [20, 45–47]. Clustering of metabolic risk factors, including central obesity, hyperglycemia, hypertriglyceridemia, and hypertension, forms the basis of the metabolic syndrome, which increases cardiovascular risk in a graded fashion [19, 48–50]. Surrogate markers of subclinical atherosclerosis, including carotid intima–media thickness and coronary artery calcium, rise progressively with the number and severity of such risk factors and are particularly elevated among individuals with combined hypertension and hyperglycemia [51–53].

Mechanistically, insulin resistance, visceral adiposity, and low-grade systemic inflammation accelerate endothelial dysfunction and vascular remodeling long before overt diabetes develops [54, 55]. Longitudinal metabolic trajectories further demonstrate that early microalbuminuria and progressive insulin resistance precede macrovascular complications, often years before clinical threshold disorders are diagnosed [48, 56, 57]. Our data align with this trajectory: prediabetes was independently associated with CMVD, and patients with combined CMVD and prediabetes exhibited markedly lower rates of functional independence. Together, these observations highlight a metabolically driven vascular vulnerability phenotype characterized by diffuse arterial injury, impaired vascular reserve, and reduced resilience to ischemic stress.

### CMVD reflects systemic rather than focal vascular vulnerability

The concept that CMVD represents systemic vascular dysfunction rather than isolated focal plaque pathology is supported by substantial literature from coronary microvascular disease (CMD). Patients with CMD frequently exhibit endothelial dysfunction and impaired vasoreactivity across peripheral vascular beds, including digital arteries, renal microcirculation, and cerebral small vessels, indicating that microvascular pathology in one organ is often mirrored systemically [58–60]. Shared mechanisms, endothelial injury, inflammation, impaired nitric oxide signaling, and altered smooth muscle reactivity operate across arterial territories and contribute to a generalized vascular instability [61–65].

Our findings extend this systemic perspective to the macrovascular level: CMVD co-segregated with coronary artery disease and metabolic dysfunction, was associated with reduced early neurological recovery, and displayed the strongest associations in embolic stroke phenotypes (cardioembolic stroke and ESUS) [16, 17]. These patterns suggest that CMVD is not merely a structural bystander but a marker of diffuse vascular fragility that constrains cerebrovascular reserve and increases susceptibility to ischemic injury. In contrast, associations between CMVD and outcome were less pronounced in small-vessel occlusion and transient ischemic attack. These findings should be interpreted cautiously given the smaller subgroup sizes and corresponding reduction in statistical power. In addition, the biological contribution of non-stenotic macrovascular abnormalities may differ across stroke mechanisms. Small-vessel occlusion is primarily driven by intrinsic microangiopathic processes, whereas transient ischemic attack often reflects transient ischemia with lower structural injury burden, potentially attenuating the measurable impact of covert macrovascular pathology on early functional outcome.

### Clinical relevance of non-stenotic carotid plaques

Our vessel-level analyses reinforce emerging evidence that non-stenotic carotid plaques (< 50% stenosis) are clinically meaningful and may contribute to stroke risk independent of luminal narrowing. Systematic reviews and multimodality imaging studies demonstrate that such plaques are highly prevalent in acute ischemic stroke and ESUS and are more often located ipsilateral to the index event, particularly when they exhibit morphological features of vulnerability, such as intraplaque hemorrhage, lipid-rich necrotic core, ulceration, surface irregularity, or positive remodeling [3–5, 35, 66, 67]. Vulnerable plaque features markedly increase the risk of recurrent stroke and TIA, with event rates approaching 4.9 per 100 person-years in the presence of intraplaque hemorrhage [10, 68, 69].

Importantly, plaque composition, rather than the degree of luminal narrowing, has emerged as the key determinant of embolic potential [10, 15, 70]. Interestingly, although the prevalence of CMVD and non-stenotic carotid disease differed substantially across stroke subtypes, high-risk plaque features themselves did not significantly differ between groups. This observation may suggest that the overall burden and multiterritorial distribution of covert macrovascular disease, rather than isolated vulnerable plaque morphology alone, contributes to cerebrovascular vulnerability and embolic risk. Alternatively, limited statistical power and incomplete high-resolution plaque characterization across imaging modalities may have reduced the ability to detect subtle subtype-specific morphological differences.

Despite acknowledgment in major vascular guidelines that plaque morphology contributes to risk [28, 71], current clinical practice remains centered on stenosis thresholds. Our findings that non-stenotic carotid plaques were independently associated with worse early outcomes and higher odds of ipsilateral stroke across embolic phenotypes reinforce the need to incorporate plaque biology and distribution into stroke evaluation and secondary prevention strategies.

### Functional and clinical implications

CMVD was associated with higher stroke severity at presentation and poorer functional recovery, independent of age, sex, stroke subtype, and major vascular comorbidities. Although the prognostic impact of CMVD was modest compared with established determinants such as baseline NIHSS, the persistence of the association after multivariable adjustment suggests that covert macrovascular pathology provides incremental information regarding vascular vulnerability and recovery potential beyond acute stroke severity alone. Diffuse macrovascular disease may impair collateral recruitment, reduce vascular reactivity, and reflect widespread endothelial dysfunction; all mechanisms that diminish cerebrovascular resilience and heighten susceptibility to ischemic injury. These patterns parallel findings from covert microvascular disease research demonstrating that structural burden in the cerebral small-vessel network predicts poorer recovery and functional decline [2].

Recent cardiovascular prevention guidelines further support the concept that subclinical and non-obstructive atherosclerotic burden carries important prognostic implications beyond conventional stenosis-based definitions. The 2026 ACC/AHA Guideline on the Management of Dyslipidemia emphasizes the growing relevance of subclinical atherosclerosis, plaque burden, and multiterritorial vascular disease for individualized cardiovascular risk stratification and implementation of intensive guideline-directed prevention strategies, even in the absence of hemodynamically significant stenosis [72]. In this context, our findings suggest that covert macrovascular pathology in stroke may represent a cerebrovascular analogue of systemic subclinical atherosclerosis, characterized by diffuse vascular vulnerability rather than isolated focal obstruction. However, our data do not support CMVD-specific treatment algorithms at present, and prospective studies are required to determine whether CMVD-guided prevention strategies improve clinical outcomes.

Clinically, the routine identification of CMVD through carotid duplex ultrasonography, CTA, MRA, or aortic imaging may provide an opportunity for refined risk stratification. Beyond general vascular risk factor modification, the identification of CMVD may support more individualized secondary prevention approaches, including intensified lipid-lowering therapy, closer metabolic optimization, and prolonged cardiac rhythm monitoring in embolic stroke phenotypes. These findings support a more holistic vascular assessment strategy beyond stenosis severity alone, although prospective studies are needed before CMVD-guided management can be formally recommended.

## Limitations

This study has several limitations. First, its single-center design may limit generalizability to broader and more diverse stroke populations. Although vascular imaging was performed systematically, high-resolution plaque characterization, particularly features such as intraplaque hemorrhage, lipid-rich necrotic core, or fibrous cap morphology, was not uniformly available across modalities, which may have led to underestimation of vulnerable plaque phenotypes. In addition, CMVD represents a heterogeneous composite phenotype comprising both atherosclerotic and structural vascular abnormalities. Although non-stenotic carotid plaque appeared to be the dominant component and showed the strongest association with unfavorable outcome, the study was not powered to fully disentangle the prognostic contribution of less frequent lesion types, including aortic arch atheroma or non-hemodynamically relevant elongation/kinking. Similarly, subgroup analyses in patients with small-vessel occlusion and transient ischemic attack were limited by smaller sample sizes and may therefore have been underpowered to detect modest subtype-specific associations.

Measurement of aortic arch atheroma, particularly for smaller plaques close to the 2-mm threshold, may be affected by inter-observer variability and imaging-modality differences. Although lesions were adjudicated by blinded, experienced investigators and borderline findings were resolved by consensus, formal reproducibility metrics were not available. Therefore, misclassification of small aortic plaques cannot be fully excluded.

Additionally, the observational nature of the study precludes causal inference, and residual confounding cannot be fully excluded despite multivariable adjustment. Although follow-up data on recurrent ischemic stroke were available, long-term outcome assessment was limited in scope. Specifically, systematic evaluation of post-stroke cognitive decline, recurrent vascular events beyond ischemic stroke, and long-term functional trajectories was not performed, which restricts conclusions regarding the broader prognostic impact of CMVD over time. Finally, metabolic variables were assessed at admission and may not fully capture longitudinal trajectories of cardiometabolic dysfunction or treatment-related changes during follow-up. Despite these limitations, the prospective design, blinded adjudication of vascular lesions, and comprehensive metabolic profiling strengthen the internal validity of the findings and support the clinical relevance of covert macrovascular disease in acute cerebrovascular events.

## Conclusions

CMVD was common in this prospective stroke cohort and was independently associated with greater stroke severity at presentation and poorer early functional outcomes. The association was most pronounced in embolic stroke phenotypes, supporting the concept that diffuse macrovascular pathology reflects systemic vascular vulnerability rather than an incidental imaging finding. These results highlight that plaque biology, rather than stenosis severity alone, contributes to early cerebrovascular risk. Incorporating covert macrovascular pathology into routine stroke evaluation may improve risk stratification and help identify patients who could benefit from intensified secondary prevention.

## Data Availability

The datasets analyzed during the current study are not publicly available due to institutional data protection regulations but are available from the corresponding author upon reasonable request and after approval by the institutional data protection officer.
